# Association between serum angiopoietin-2 concentrations and periprocedural myocardial injury in patients undergoing elective percutaneous coronary intervention

**DOI:** 10.18632/aging.102936

**Published:** 2020-03-17

**Authors:** Wen Jian, Jia-Hui Guan, Wen-Bo Zheng, Chang-Hua Mo, Yu-Tao Xu, Qi-Li Huang, Chun-Mei Wei, Can Wang, Zhi-Jie Yang, Guo-Liang Yang, Chun Gui

**Affiliations:** 1 Department of Cardiology, The First Affiliated Hospital of Guangxi Medical University, Nanning 530021, Guangxi, People's Republic of China; 2 Guangxi Key Laboratory Base of Precision Medicine in Cardio-Cerebrovascular Diseases Control and Prevention, Nanning 530021, Guangxi, People's Republic of China; 3 Guangxi Clinical Research Center for Cardio-Cerebrovascular Diseases, Nanning 530021, Guangxi, People's Republic of China; 4 Department of Respiratory Medicine, The First Affiliated Hospital of Guangxi Medical University, Nanning 530021, Guangxi, People’s Republic of China

**Keywords:** angiopoietin-2, perioperative myocardial injury, coronary artery disease, percutaneous coronary intervention, NT-proBNP

## Abstract

Angiopoietin-2 (Ang-2) is a proangiogenic factor that mediates inflammation and atherosclerosis. We evaluated the predictive value of circulating Ang-2 levels for periprocedural myocardial injury (PMI) in 145 patients undergoing elective percutaneous coronary intervention (PCI), and investigated whether post-PCI Ang-2 levels are influenced by PMI. PMI was defined as a post-procedural troponin elevation above the 5×99^th^ percentile upper reference limit. Blood samples for Ang-2 analysis were collected at admission and on postoperative days 1 and 3. PMI occurred in 40 patients (28%). At baseline, there was no difference in Ang-2 levels between PMI and non-PMI patients (P=0.554). However, a significant interaction effect between PMI occurrence and time on Ang-2 levels was observed (interaction P=0.036). Although serum Ang-2 levels in non-PMI patients gradually decreased, Ang-2 levels in PMI patients did not change between different time-points. Multiple logistic regression analysis revealed that age, total stent length, and serum levels of N-terminal pro-brain natriuretic peptide were independent PMI predictors. These findings indicate that pre-procedural Ang-2 levels do not impact PMI occurrence after elective PCI. However, changes in Ang-2 levels after the procedure are closely related to PMI.

## INTRODUCTION

Percutaneous coronary intervention (PCI) has become an effective and safe method for revascularization in patients with coronary artery disease (CAD), especially in an elective setting [1]. However, the high incidence of periprocedural myocardial injury (PMI) is of great concern as emerging evidence suggests a link between PMI and mortality [2–4]. PMI is frequently attributed to myocardial damage via distal embolization, side-branch occlusion, and other unexpected procedural complications. As such, PMI is most likely to occur in patients with advanced lesions, complex anatomy, and difficult procedures [5].

Angiopoietins (Ang) are a family of growth factors that consist of four members: Ang-1, Ang-2, Ang-3, and Ang-4 [6]. Whereas Ang-1 maintains endothelial quiescence and integrity through binding the ligand of Tie2 receptor, Ang-2 acts as a context-dependent Tie2 antagonist, which inhibits the Ang-1-induced Tie2 phosphorylation, thus impairing endothelial integrity and promoting vascular leakage [7]. Ang-2 can be rapidly released from Weibel-Palade bodies of endothelial cells upon stimulation, such as hypoxia and inflammation [8, 9]. As an important proangiogenic factor, Ang-2 has been recently implicated in mediating inflammation [10–12] and atherosclerosis [13, 14]. Ang-2 is abundantly expressed in advanced human atherosclerotic lesions, and is associated with microvessel density [15, 16]. In patients with CAD, especially acute myocardial infarction (AMI), the circulating Ang-2 levels are significantly increased [17].

Previous studies have indicated an association between lesion-related risk factors and PMI [18]. The morphological characteristics of a vulnerable plaque phenotype, determined by intravascular imaging modalities, can predict the PMI occurrence [19–21]. Given the close relationship between Ang-2 levels, inflammation, and atherosclerosis, it is important to determine whether the circulating Ang-2 levels can predict the occurrence of PMI. Furthermore, previous studies have shown a significant association between mortality and circulating Ang-2 levels measured over time in multiple clinical scenarios [22–24]. We hypothesize that an increase in the circulating Ang-2 levels during hospitalization might be partly related to prominent myocardial injury, which contributes to poorer outcomes. Thus, this study aimed to evaluate the predictive value of circulating Ang-2 levels for PMI occurrence, and to investigate whether the changes in Ang-2 levels after PCI are influenced by PMI.

## RESULTS

### PCI patients’ characteristics

**Figure 1:**
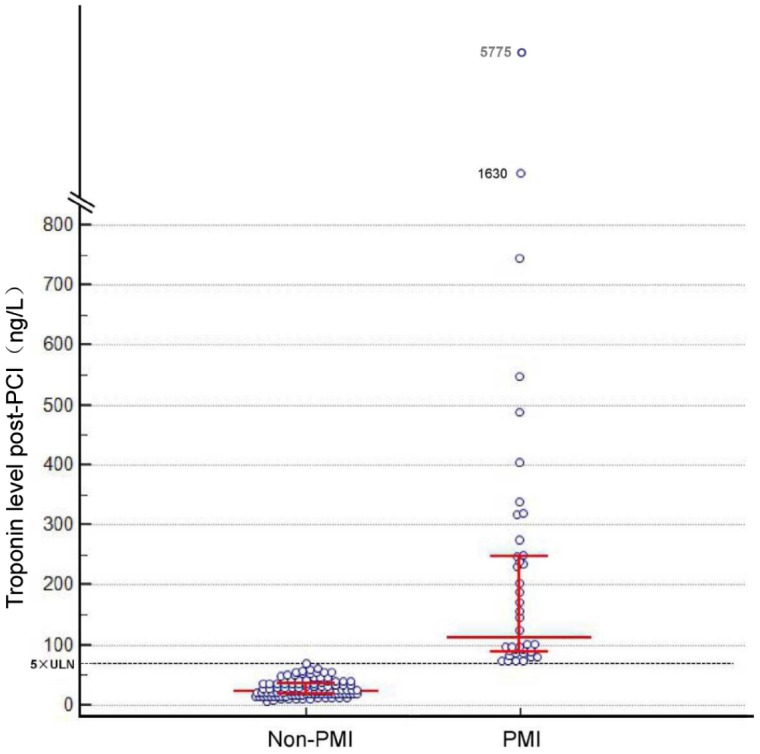
**Post-percutaneous coronary intervention troponin levels.** Bars indicate median levels with interquartile range. The dotted line indicates the limit of 70 ng/L. ULN, upper limit of the normal.

From 145 patients who underwent elective PCI, PMI occurred in 40 patients (28%). The patients’ baseline clinical characteristics are detailed in Table 1. Table 2 summarizes the angiographic and procedural characteristics. The mean age of the patients was 61 ± 10 years; 83% were men. Patients with PMI were older, and had higher serum levels of N-terminal pro-brain natriuretic peptide (NT-proBNP) compared to non-PMI patients (Table 1). There was no difference in demography, medical history, medications, and baseline laboratory data between the two groups (Table 1). Figure 1 shows the post-procedural levels of high-sensitivity troponin T (hsTnT) in PMI and non-PMI patients. After PCI, hsTnT was higher in PMI patients than in non-PMI patients, while there was no significant difference in serum creatinine (Table 1). Of note, regarding to the procedural characteristics, patients with PMI had higher SYNTAX (Synergy Between Percutaneous Coronary Intervention With Taxus and Cardiac Surgery) scores, and more frequently received longer stent placements compared with non-PMI patients (Table 2).

**Table 1:** Patients’ characteristics.

	**All patients (n=145)**	**PMI group (n=40)**	**Non-PMI group (n=105)**	***P*-value**
Demography and medical history				
Age	61 ± 10	64 ± 10	60 ± 9	0.008
BMI	24.6 ± 3.6	24.0 ± 4.2	24.9 ± 3.4	0.170
Male gender	120 (83%)	34 (85%)	86 (82%)	0.659
Smoking	71 (49%)	18 (45%)	53 (50%)	0.555
Hypertension	103 (71%)	30 (75%)	73 (70%)	0.516
Diabetes	52 (36%)	17 (43%)	35 (33%)	0.304
Renal dysfunction	17 (12%)	6 (15%)	11 (11%)	0.640
Previous PCI	51 (35%)	12 (30%)	39 (37%)	0.421
Chronic heart failure	36 (25%)	13 (33%)	23 (22%)	0.187
Cerebrovascular disease	28 (19%)	8 (20%)	20 (19%)	0.897
Medications				
Statin	142 (98%)	39 (98%)	103 (98%)	1.00
Aspirin	140 (97%)	38 (95%)	102 (97%)	0.902
Clopidogrel	98 (68%)	27 (68%)	71 (68%)	0.989
Ticagrelor	44 (30%)	13 (33%)	31 (30%)	0.728
Glycoprotein IIb/IIIa inhibitor use	23 (16%)	5 (13%)	18 (17%)	0.494
Echocardiographic result				
LVEF (%)	66 (61–71)	66 (57–70)	66 (62–71)	0.438
Laboratory tests at admission				
HbA1_c_ (%)	6.2 (5.8–6.8)	6.2 (5.8–6.8)	6.2 (5.8–6.8)	0.712
Total cholesterol (mmol/L)	4.3 ± 1.0	4.3 ± 0.8	4.2 ± 1.1	0.642
LDL cholesterol (mmol/L)	2.3 ± 0.9	2.4 ± 0.8	2.3 ± 1.0	0.626
WBC (×10^9^/L)	6.9 ± 1.8	6.7 ± 1.6	7.0 ± 1.9	0.401
NLR	2.4 ± 1.1	2.3 ± 1.1	2.4 ± 1.2	0.645
NT-proBNP (pg/ml)	121 (50–423)	245 (69–673)	88 (50–271)	0.011
Creatinine (umol/L)	87 ± 21	88 ± 23	86 ± 20	0.691
hsTnT (ng/L)	9 (6–12)	10 (6–12)	9 (6–11)	0.285
Ang-2 (Log) at the baseline	3.18 ± 0.28	3.20 ± 0.35	3.17 ± 0.25	0.554
Laboratory tests post-PCI				
Creatinine (umol/L)	92 ± 24	92 ± 25	91 ± 24	0.855
hsTnT (ng/L)	35 (19–78)	135 (89–269)	24 (18–37)	<0.001
Ang-2 (Log) on postoperative day 1	3.14 ± 0.26	3.17 ± 0.26	3.12 ± 0.26	0.296
Ang-2 (Log) on postoperative day 3	3.11 ± 0.28	3.19 ± 0.26	3.09 ± 0.28	0.051

Data are presented as mean ± SD, or median (IQR, interquartile range), or number (percentage). Abbreviations: Ang-2=angiopoietin-2; BMI=body mass index; BP=blood pressure; HbA1_c_=hemoglobin A1c; hsTnT=high sensitive troponin-T; LDL=low-density lipoprotein; LVEF=left ventricular ejection fraction; NLR=neutrophil-to-lymphocyte ratio; NT-proBNP=N-terminal pro-brain natriuretic peptide; PCI=percutaneous coronary intervention; WBC=white blood cell.

**Table 2:** Procedural Characteristics.

	**All patients (n=145)**	**PMI group (n=40)**	**Non-PMI group (n=105)**	***P*-value**
SYNTAX score	16 ± 10	19 ± 10	15 ± 9	0.019
Description of the target lesions				
Multiple vessels (two or more)	24 (17%)	7 (18%)	17 (16%)	0.850
LM	8 (6%)	2 (5%)	6 (6%)	1.000
LAD	74 (51%)	22 (55%)	52 (50%)	0.555
LCx	36 (25%)	14 (35%)	22 (21%)	0.080
RCA	55 (38%)	11 (28%)	44 (42%)	0.110
CTO	14 (10%)	5 (13%)	9 (9%)	0.688
PTCA without stenting	8 (6%)	1 (3%)	7 (7%)	0.565
Number of stents	1.4 ± 0.6	1.6 ± 0.6	1.3 ± 0.6	0.004
Total stent length, mm	35 ± 21	44 ± 21	32 ± 20	0.003
Pre-dilation	139 (96%)	39 (98%)	100 (95%)	0.885
Post-dilation	127 (88%)	37 (93%)	90 (86%)	0.409
Max balloon pressure (atm)	17.5 ± 3.8	17.7 ± 3.7	17.4 ± 3.9	0.625
Post-PCI TIMI-3 flow	144 (99%)	39 (98%)	105 (100%)	0.276

Abbreviations: CTO=chronic total occlusion; LAD=left anterior descending coronary artery; LM=left main coronary artery; LCx=left circumflex coronary artery; PTCA=percutaneous transluminal coronary angioplasty; RCA=right coronary artery; SYNTAX=Synergy Between Percutaneous Coronary Intervention With Taxus and Cardiac Surgery.

### Association between circulating Ang-2 levels and PMI

**Figure 2:**
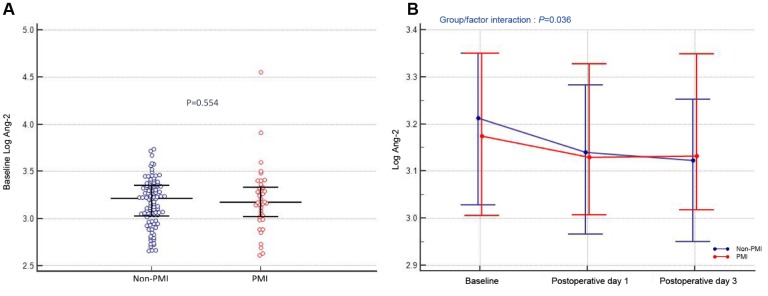
**Association between circulating Ang-2 levels and PMI.** (**A**) Preoperative angiopoietin-2 levels (Log). (**B**) Angiopoietin-2 levels (Log) at different time-points (at admission, postoperative day 1 and day 3) in patients stratified by PMI. The dots indicate median levels, and bars represent interquartile range.

At baseline, there was no difference in Ang-2 levels between PMI and non-PMI patients (Log: 3.20 ± 0.35 vs. 3.17 ± 0.25, P=0.554; Figure 2A). However, a significant interaction effect between PMI occurrence and time on Ang-2 levels was observed (interaction P=0.036; Figure 2B). Serum Ang-2 levels in non-PMI patients gradually decreased after PCI (Log: 3.17 ± 0.25 vs. 3.12 ± 0.26 vs. 3.09 ± 0.28, P<0.001), whereas there was no change in Ang-2 levels after PCI in PMI patients (Log: 3.20 ± 0.35 vs. 3.17 ± 0.26 vs. 3.19 ± 0.26, P=0.442; Figure 2B).

### Predictive factors analysis of PMI

**Figure 3:**
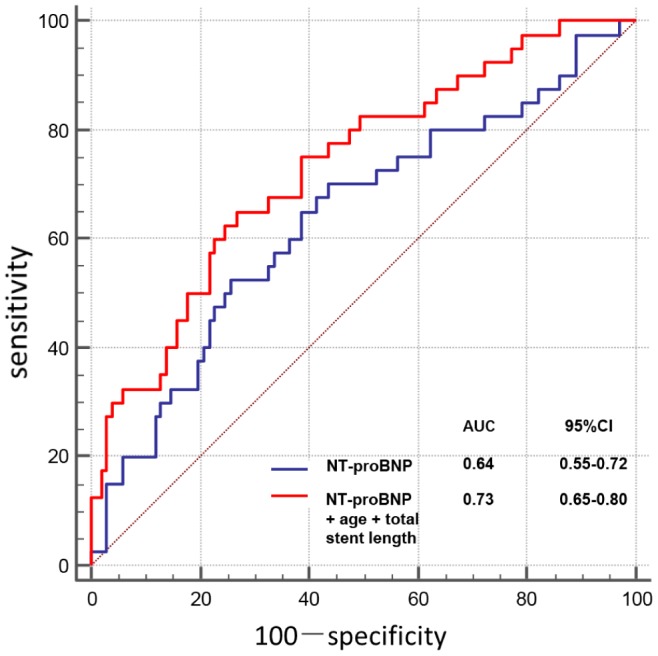
**Receiver operating characteristic curve for relevant variables predicting PMI.**

As illustrated in Figure 3, receiver operating characteristic (ROC) curve analysis showed that NT-proBNP predicted PMI with 53% sensitivity and 74% specificity, using an optimal cutoff of 213 pg/ml (area under curve [AUC], 0.64; 95% confidence interval [CI], 0.55-0.72; P=0.011). In multivariable logistic regression analysis, age (per 10 years, odds ratio [OR]: 2.08; 95% CI: 1.25–3.48; P=0.005), total stent length (per 10 mm, OR: 1.36; 95% CI: 1.09–1.70; P=0.007), and NT-proBNP >213 pg/ml (OR: 2.63; 95% CI: 1.08–6.38; P=0.032) were independent predictors of PMI (Table 3). Combination of these 3 characteristics showed a high predictive value (AUC, 0.73; 95% CI, 0.65-0.80; P<0.001; Figure 3).

**Table 3:** Binary logistic regression model in univariate and multivariate analysis for predicting PMI.

	**Univariable**			**Multivariable**	
	**OR (95% CI)**	**P value**		**OR (95% CI)**	**P value**
Age, per 10 years	1.75 (1.15–2.68)	0.010		2.08 (1.25–3.48)	0.005
SYNTAX score, per 10 points	1.55 (1.07–2.26)	0.022		1.12 (0.69–1.83)	0.636
Total stent length, per 10 mm	1.31 (1.10–1.58)	0.003		1.36 (1.09–1.70)	0.007
NT-proBNP (>213 pg/ml)	3.19 (1.49–6.85)	0.003		2.63 (1.08–6.38)	0.032

Notes: The multivariate model included age, gender, hypertension, diabetes, renal dysfunction, SYNTAX score, multivessel lesion, left main artery lesion, left anterior descending artery lesion, total stent length, and NT-proBNP. CI=confidence interval; NT-proBNP=N-terminal pro-brain natriuretic peptide; OR=odds ratio; PMI=periprocedural myocardial injury; SYNTAX=Synergy Between Percutaneous Coronary Intervention With Taxus and Cardiac Surgery.

## DISCUSSION

This study is the first to describe the relationship between serum Ang-2 levels and PMI occurrence in patients undergoing elective PCI. The major findings are: (1) There is no association between preoperative Ang-2 levels and PMI occurrence; (2) after PCI, the change of Ang-2 levels is closely related to PMI; and (3) NT-proBNP is an independent predictor of PMI.

In previous studies, PMI has been reported to be patient-, lesion-, and procedure-related [18]. An increase in cardiac biomarkers post procedure is very common for patients undergoing PCI [25], and the incidence of PMI can vary remarkably from 5 to 33%, depending on the cardiac biomarker, marker assay, and the criteria of cut-off value [26, 27]. Notably, probably because of the use of high-sensitivity troponin assays, and the lack of effective preventive strategies [28, 29], we observed a high PMI rate of 28%. Since our study did not observe fatal or severe complications, such as acute stent thrombosis, bleeding, perforation, or stroke during the procedure, we assume that a large proportion of PMI may be attributed to the distal embolization of plaque debris.

Plaque composition, verified by multiple intravascular imaging modalities, such as optical coherence tomography [19], intravascular ultrasound [20], or near infrared spectroscopy [21], is a predominant contributor to cardiac enzyme elevation post-PCI. Several inflammatory biomarkers have been shown to predict PMI occurrence, though the underlying mechanisms are not understood [30, 31]. We assume that the predictive value of these biomarkers for PMI may not be solely based on a direct influence of the systemic inflammatory state but reflect a parallel activation of related factors and the plaque status. Our study indicates that the pre-procedural serum Ang-2 levels do not affect the occurrence of PMI. One possible explanation is that even though Ang-2 plays a pivotal role in the inflammatory response, the circulating Ang-2 levels may not be a good biomarker reflecting the plaque status. Ang-2 expression is low in quiescent endothelium, but is upregulated following endothelial activation by stress, hypoxia, and pro-inflammatory factors [8–10]. Previous studies have demonstrated that Ang-2 is abundantly expressed locally in advanced lesions [15, 16]. However, it remains unclear whether the circulating Ang-2 levels are related to unstable plaque features, such as plaque volume, lipid core, or fibrous cap thickness. Lee et al. reported no difference in plasma Ang-2 levels between patients with unstable and stable angina [17]. Charytan et al. recently found that, in non-AMI patients, there was no significant association between serum Ang-2 levels and the total burden of coronary plaque [32]. Similar results were also described by Mitsuma et al., who found that serum Ang-2 levels were not significantly different between mild and severe CAD groups (classified by maximal percent stenosis) [33]. Given the fact that our study subjects were in a non-acute condition and scheduled for elective procedure, the relatively low circulating Ang-2 levels, which were insensitive for the discrimination of vulnerable coronary plaque, might have contributed to the negative results in this study. Since the circulating Ang-2 levels are influenced by many factors, including cardiac function [34], renal function [35], and various inflammatory conditions [11], the predictive efficiency of Ang-2 for PMI might be compromised. In addition, in this study, we used high-sensitivity troponin assays that allowed detection of a minuscule damage to myocardial tissue. Given the use of 5-fold hsTnT criteria in this study, the different cut-off values and other cardiac biomarkers, such as creatine kinase-MB, should be investigated in the future.

Importantly, our study indicates that the level of NT-proBNP, a marker reflecting pressure and volume load on the heart, is an independent predictor of PMI. This finding is consistent with a previous study which showed that the NT-pro-BNP level is a useful biomarker for predicting PMI in diabetic patients without cardiac dysfunction [36]. Although NT-proBNP is not a direct indicator of necrosis, NT-proBNP characterizes the functional state of the myocardium. Previous studies have shown that the high levels of NT-proBNP are associated with myocardial ischemia [37], and the magnitude of NT-proBNP increase after PCI correlates with the extent of myocardial injury [38]. However, we failed to provide an explanation regarding the association of preoperative NT-proBNP levels with PMI occurrence. Given the multiple clinical application values of NT-proBNP in different scenarios beyond heart failure [34, 39, 40], further large studies need to be conducted to explore and verify its precise cut-off point in predicting PMI.

Alleviation of myocardial ischemia and the effects of drugs, such as statins [41], may explain the decrease in Ang-2 levels in day 1 samples compared to the admission samples. This trend even remains in patients with PMI, although it did not reach a statistical difference. Of note, the fall in Ang-2 levels was followed by a significant increase in day 3 in PMI patients, whereas in non-PMI patients, the falling trend continued. Increased Ang-2 levels measured over time have been established as an important prognostic factor in various diseases [22–24]. We have recently found that Ang-2 levels post-PCI, but not pre-PCI, can predict the occurrence of adverse cardiovascular events [42]. However, it remained unknown whether circulating Ang-2 levels after PCI are influenced by PMI. Therefore, this study focused on a well-characterized study population with negative troponin at admission to assess the association between Ang-2 and PMI. Intriguingly, the results are in line with our hypothesis that PMI can increase circulating Ang-2 levels. We assume that this phenomenon could be explained by microembolization. It has been previously suggested that the extent of troponin elevation after PCI correlates with a new, irreversible myocardial necrosis [43], which can induce local myocardial inflammation. Fam et al. [44] showed that Ang-2 is one of the most upregulated genes in biopsies of ischemic myocardium in patients with acute coronary syndrome. In a mouse infarct model, there was a significant gradual increase of Ang-2 expression by endothelial cells occurring at the infarct border zone, and this increase was maintained until day 7. Likewise, in a model of ischemia/reperfusion injury, Ang-2 expression began rising on day 2, but waned beginning on day 3 [45]. Therefore, the myocardial damage by microembolization due to plaque debris may increase the expression of Ang-2 in endothelial cells residing at the damaged border zone and influence its circulating levels. Thus, our data indicate that myocardial injury, reflected by increased troponin levels, is an important contributor to the elevation of circulating Ang-2 levels. This marked association can partly explain the recent evidence regarding the Ang-2 prognostic values.

An excessive and uncontrolled inflammation can exacerbate myocardial damage following PCI. Ang-2 can prime the inflammatory response, sensitize endothelial cells toward tumor necrosis factor-α, and increase expression of adhesion molecules, resulting in increased leukocyte recruitment [9]. Furthermore, Ang-2 can act as a paracrine chemoattractant promoting migration of innate immune cells by binding Tie2 receptors or in an integrin-dependent manner [12, 46, 47]. Whereas Ang-2 overexpression can facilitate the responsiveness of acute inflammation [12], Ang-2 downregulation can ameliorate post-ischemic cardiovascular remodeling after myocardial infarction, and attenuate myocardial death after ischemia/reperfusion injury [45]. Thus, the increased Ang-2 release from the heart after PCI can further aggravate the myocardial injury by amplifying cardiac hypoxia and inflammation.

Several limitations should be considered when interpreting our findings. First, this study was a single-center observational study with a relatively small sample size; thus, it is reasonable to assume that a certain amount of selection bias may exist in our study. Second, the post-procedural evidence of new myocardial ischemia, which is indispensable for the diagnosis of “type 4a myocardial infarction” [48], was not considered in this study. Third, since we collected the fasting blood samples in the morning on postoperative days 1 and 3, the extraction time was not strictly controlled. More data and large-scale studies are needed in the future.

Collectively, our findings demonstrate that the pre-procedural Ang-2 levels do not impact the PMI occurrence after elective PCI. However, the changes in Ang-2 levels after the procedure are closely related to PMI. Targeting the Ang-2 pathway may serve as a potential novel strategy for reducing the occurrence of PMI.

## MATERIALS AND METHODS

### Study population

The study enrolled 145 consecutive patients who underwent elective PCI with a negative hsTnT level at the time of admission, between September 2018 and September 2019. Patients were referred for numerous reasons, including atypical chest pain, stable or unstable angina, previous diagnosis of CAD in a setting of scheduled operation, or other indications for diagnostic coronary angiography. The following exclusion criteria were applied: AMI, acute heart failure, elevated hsTnT levels at admission (>99^th^ percentile upper reference limit [URL]), unavailable measurements of serial (preprocedural and postprocedural) hsTnT, tachyarrhythmia, valvular or congenital heart disease, concomitant inflammatory conditions, end-stage renal disease, malignancies, and recent surgery or trauma (within 3 months). Medications at admission and perioperative anticoagulant therapy were chosen based on the accepted guidelines and standard methods. Dual antiplatelet treatment, including aspirin, and a P2Y12 inhibitor (chosen by the interventional cardiologist) were initiated at admission, during PCI, or immediately after PCI. Unfractionated heparin was used during the operation, and the periprocedural use of glycoprotein IIb/IIIa inhibitors was left to the operator’s discretion. Drug-eluting stents were used for patients who needed stent placement. Angiographic characteristics were recorded by experienced angiographers. The study protocol was reviewed and approved by the Human Research Ethics Committee of the First Affiliated Hospital of Guangxi Medical University, China. All patients provided written informed consent.

### Related definitions

PMI was defined based on hsTnT criteria (elevation of hsTnT above 5 x 99^th^ percentile URL) occurring within 24 hours after the procedure suggested by the fourth universal definition [48]. Body mass index (BMI) was defined as the body mass divided by the square of the body height (kg/m^2^). Diabetes mellitus was defined as either a new diagnosis based on the American Diabetes Association guidelines [49] or a history of taking antidiabetic medications. Hypertension was defined as a systolic blood pressure > 140 mmHg, diastolic blood pressure > 90 mmHg, or a history of taking antihypertensive medications. Renal dysfunction was defined as estimated glomerular filtration rate [50] <60 mL/min/1.73 m^2^. The SYNTAX score was calculated to grade the complexity of coronary lesions [51].

### Laboratory measurements

In our hospital, screening for PMI and contrast-induced nephropathy was routinely performed after the PCI. In brief, hsTnT levels were measured at admission, postoperative day 1, and when clinically indicated. Furthermore, repeated measurement of serum creatinine was conducted on postoperative day 3. Fasting blood samples for Ang-2 analysis were collected under standardized conditions at admission and on postoperative days 1 and 3. HsTnT was measured by electrochemiluminescence immunoassay (Roche Diagnostics, Risch-Rotkreuz, Switzerland) (99^th^ percentile URL: 14 ng/L). Serum Ang-2 levels were measured using commercially available enzyme-linked immunosorbent assay kits (RayBiotech, Inc, Norcross, GA, USA). The limit of detection for Ang-2 was 10 pg/ml, with the intra-assay variability <10 % and the inter-assay variability <12 %.

### Statistical analysis

Continuous variables are presented as mean ± SD or median (IQR, interquartile range), and were compared using Student t test or Mann-Whitney U test, as appropriate. Categorical variables are presented as percentage of patients; they were compared by chi-square or Fisher exact test. As the distribution of Ang-2 was skewed, a base-10 logarithmic transformation was applied. A 2-factor mixed ANOVA was used to test the interaction effect between PMI and time on Ang-2 levels after the procedure. One-way repeated-measures ANOVA test was used to analyze the Ang-2 levels over time in each group. ROC curve analysis was performed to evaluate the diagnostic value of relevant variables for PMI. Multivariable logistic regression model was constructed to assess the independent predictors of PMI, including the variables that were selected based on clinical judgement, or the known risk factors based on literature review [18], or P value < 0.05 in the univariate analysis. Ultimately, age, gender, hypertension, diabetes, renal dysfunction, SYNTAX score, multivessel lesion, left main artery lesion, left anterior descending artery lesion, total stent length, and NT-proBNP were included in the multivariate model. A 2-tailed *P* value < 0.05 was considered significant. Statistical analysis was performed using SPSS, version 22 and MedCalc, version 19.0.4.
